# Toxic Megacolon With a Positive Clostridioides difficile Antigen but Negative Toxin Assay

**DOI:** 10.7759/cureus.103324

**Published:** 2026-02-09

**Authors:** Milikyas A Feyisa

**Affiliations:** 1 General Internal Medicine, Naas General Hospital, Naas, IRL

**Keywords:** c. difficle antigen positive, c. difficle toxin negative, eia, proton pump inhibitors (ppi), toxic megacolon

## Abstract

The diagnosis of *Clostridioides difficile* infection (CDI) relies on a combination of clinical presentation and laboratory testing. However, a critical diagnostic discordance occurs when laboratory results contradict a rapidly evolving clinical picture. This is a case of a 78-year-old man with four comorbidities who presented with watery diarrhea and a *C. difficile* screen-positive but toxin-negative stool sample. His main risk factors included advanced age and the prolonged use of a proton pump inhibitor. Despite his initial negative toxin assay, his condition progressively worsened over three days following his admission, with the development of toxic megacolon, diagnosed clinically and radiologically. This report synthesizes evidence on the limitations of toxin enzyme immunoassays in severe CDI, contextualizing a well-documented, but dangerously overlooked, phenomenon where test sensitivity fails in the sickest patients. This case highlights the imperative to treat the patient, not the lab result, when clinical and radiological evidence suggests fulminant disease.

## Introduction

*Clostridioides difficile* infection (CDI) is a leading cause of healthcare-associated diarrhea, with a spectrum ranging from asymptomatic colonization to mild colitis to life-threatening toxic megacolon and septic shock [1]. Broad-spectrum antibiotics are the main predisposing factors, while the use of gastric acid suppressants (proton pump inhibitors (PPIs) and H2 antagonists), prolonged hospitalization, and immunocompromised states (advanced age or acquired immunodeficiency) are also important risk factors [2].

Modern laboratory diagnostic algorithms often employ a two-step process using a sensitive screening test for glutamate dehydrogenase (GDH), followed by a confirmatory test for toxins A and B via enzyme immunoassay (EIA) [3]. While cost-effective, the toxin EIA has known sensitivity limitations, particularly in severe disease due to reasons discussed in this article. This limitation can cause a diagnostic challenge if sound clinical judgement is not used along with supplementary imaging. This case report illustrates the perilous clinical scenario where reliance on a negative toxin EIA can delay critical, life-saving treatment in a patient progressing to toxic megacolon.

## Case presentation

A 78-year-old man was admitted with a two-day history of fatigue, lethargy, and five episodes of watery diarrhea. He had no abdominal pain or fever spikes. His past medical history was significant for schizophrenia, a remote brain hemorrhage with craniotomy, seizure, and high cholesterol. He lived alone with home care support twice daily and used a walking stick for mobility. Regular medications included pantoprazole, aripiprazole, phenytoin, rosuvastatin, folic acid, and nutritional supplements. There was no recent history of antibiotic use or hospitalization in the preceding two years.

On admission, he was pleasantly confused (4AT score, 4/12; Glasgow Coma Scale score, 14/15) with dry mucous membranes. Abdominal examination was initially unremarkable, revealing a soft, non-tender, and non-distended abdomen. Initial blood tests revealed leukocytosis (white cell count, WCC 22.1 x 10⁹/L; neutrophils, 20.5 x 10⁹/L) and an elevated C-reactive protein (CRP) (54 mg/L). Hemoglobin level was 17 g/dL. An abdominal X-ray showed gassy bowel loops but no evidence of obstruction or free air. A stool sample was reported on the next of admission as *C. difficile* screen (GDH) was positive, but the toxin A/B EIA was negative. The stool pathogen panel is shown in Table 1.

**Table 1:** Stool pathogen panel.

Pathogen	Result
Yersinia enterocolitica	Not detected
*Shigella*/EIEC	Not detected
*Escherichia coli* O157	Not detected
*Clostridioides difficile* screen	Positive
*Clostridioides difficile* toxin	Not detected
Verotoxin gene	Not detected
Salmonella	Not detected
Campylobacter	Not detected
Ova/Parasite	Not detected
Norovirus	Not detected

**Figure 1:**
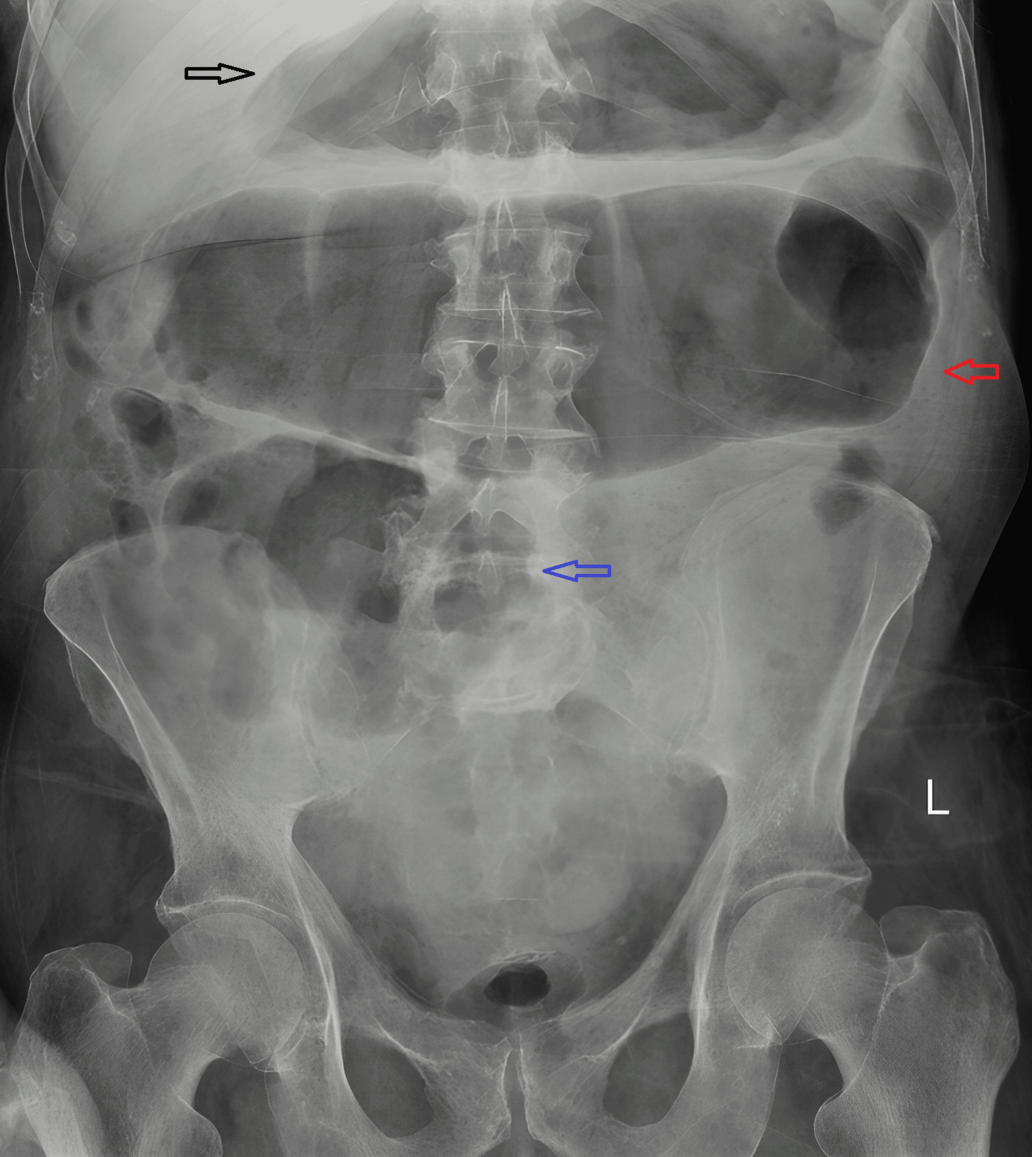
Abdominal X-ray on day three. Dilated transverse colon measuring up to 9.7 cm in keeping with megacolon (red arrow), with gassy distension of the stomach (black arrow) and the caecum (blue arrow).

**Figure 2:**
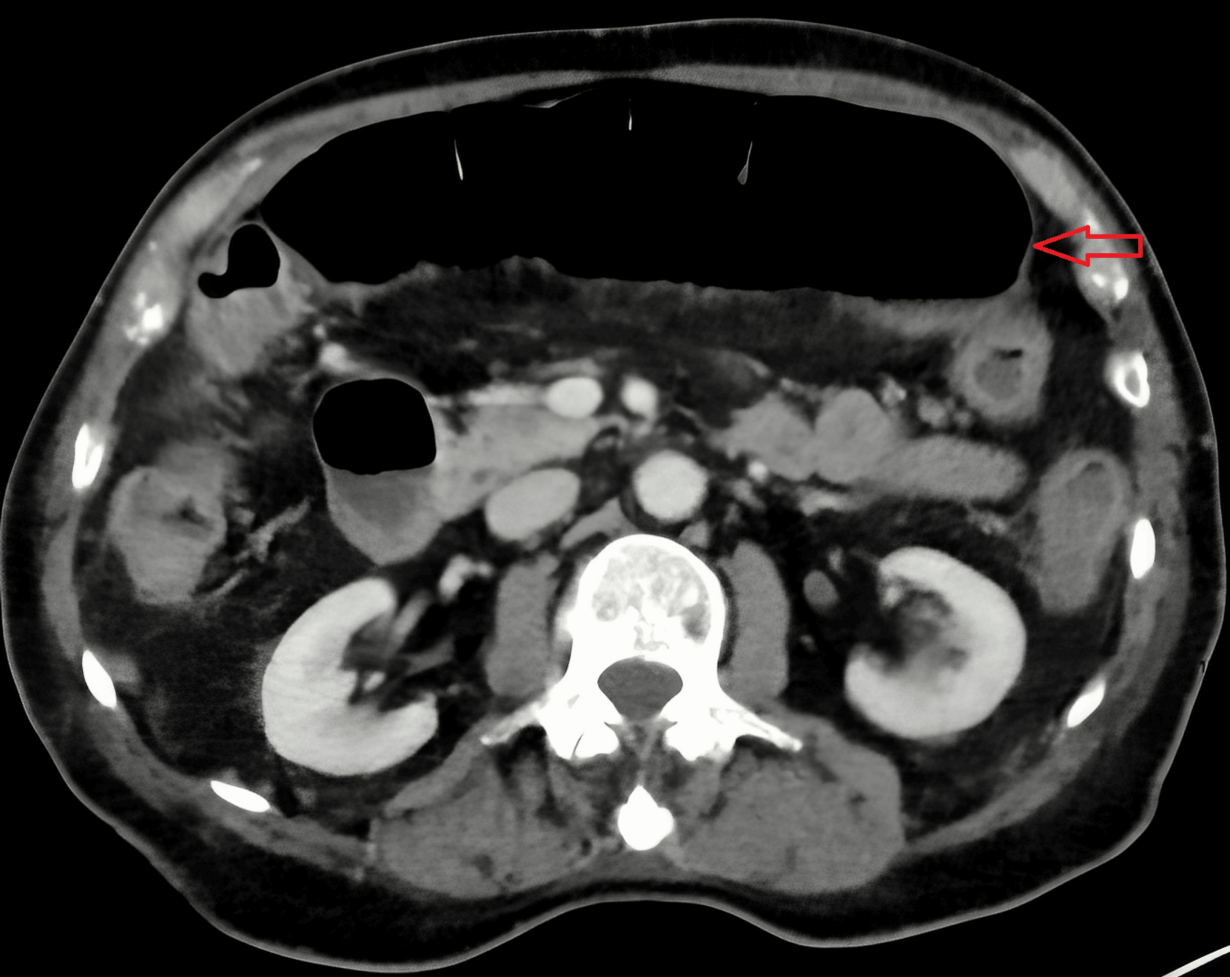
CT scan of abdomen and pelvis on day three. The transverse colon (red arrow) is dilated measuring 5.7 cm in the anteroposterior diameter and 7.7 cm in the craniocaudal diameter consistent with megacolon.

By day three of admission, the patient subjectively felt better, was eating and drinking, and had no further vomiting. However, he continued to have high-volume diarrhea (up to six times per day of type 7 diarrhea). Abdominal examination revealed new distension with hypertympany. His CRP rose sharply to 164 mg/L. A repeat abdominal X-ray demonstrated a dilated transverse colon measuring 9.7 cm, consistent with megacolon (Figure 1). Serum lactate remained normal (0.6-0.7 mmol/L). CT of the abdomen/pelvis confirmed the diagnosis (Figure 2), showing diffuse circumferential thickening and mucosal hyperenhancement of the entire colon and rectum (proctocolitis) and a dilated transverse colon (5.7-7.7 cm) with reduced wall enhancement, consistent with megacolon complicating severe colitis. The discrepancy between the radiographic (9.7 cm) and CT (5.7 cm anteroposterior and 7.7 craniocaudal) measurements is attributable to both imaging technique and pathophysiological change. Projection radiography can overestimate the diameter of a tortuous, gas-filled loop, while CT provides a precise axial measurement. Furthermore, during interval hours between the two imagings allowed for possible colonic decompression and redistribution of intraluminal content, altering the gas-to-fluid ratio seen on CT.

**Figure 3:**
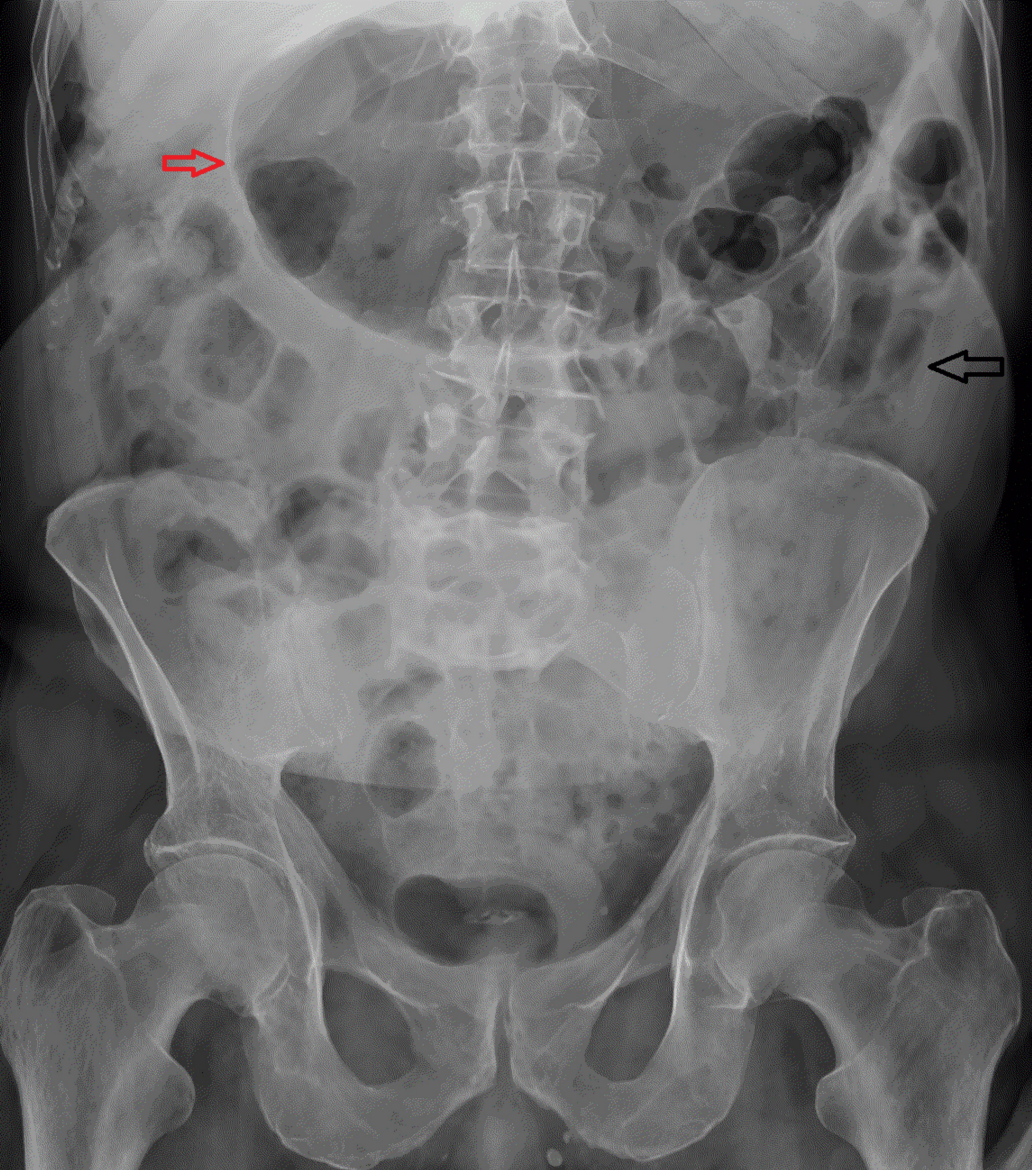
Follow-up abdominal X-ray. The black arrow shows interval improvement in the transverse colon and the red arrow shows the gassy stomach.

Given the ongoing profuse watery diarrhea and radiological findings, antibiotic therapy was started with high-dose oral vancomycin (500 mg four times daily) and intravenous metronidazole (400 mg three times daily), alongside aggressive electrolyte replacement. Surgical review deemed the patient medically unfit for operative intervention. Progressively, the diarrhea started to settle, decreasing to one to two episodes per day of type 5 stool. On the eighth day of admission, his diarrhea settled, and he was engaging well with physiotherapy to attain his baseline mobility. A follow-up abdominal X-ray showed interval improvement (Figure 3).

## Discussion

CDI is a leading cause of healthcare-associated diarrhea, with a spectrum ranging from mild colitis to life-threatening toxic megacolon and septic shock. This case exemplifies a well-documented but clinically treacherous pitfall in CDI diagnosis: the development of fulminant colitis and toxic megacolon despite an initial negative stool toxin EIA. The two-step diagnostic algorithm (GDH + toxin EIA) is widely used for its high negative predictive value and cost-effectiveness [3]. However, its weakness lies in the suboptimal sensitivity of the toxin EIA. Meta-analyses report a sensitivity of approximately 75% compared to the gold-standard cell culture cytotoxicity neutralization assay, meaning one in four true cases may yield a false-negative toxin result [4,5].

Several pathophysiological mechanisms explain this phenomenon in severe disease, which can explain our patient’s presentation. One mechanism is that the dilutional effect of a profuse, watery diarrhea leads to significant stool dilution, potentially reducing toxin concentration below the detection threshold of the EIA [6,7]. The other is toxin sequestration that occurs in fulminant colitis, where *C. difficile *toxin B (TcdB) binds extensively to high-affinity receptors (e.g., Frizzled proteins, CSPG4) on colonic epithelial cells [8-10]. This binding and subsequent internalization “sequesters” the toxin within the colonic tissue, leaving less free toxin in the stool lumen to be detected by commercial assays [8,10,11]. Lastly, patients who are immunosenescent (due to advanced age, as in this patient) might not mount an adequate response, resulting in an inflammatory cascade outpacing toxin production or release, creating a diagnostic window where stool toxin levels are low despite severe tissue damage [11].

This patient’s clinical trajectory underscores the vital recommendations from the Infectious Diseases Society of America/Society for Healthcare Epidemiology of America guidelines, which advise initiating empirical treatment for CDI when clinical suspicion is high, without waiting for test results [5]. Furthermore, in suspected severe cases, imaging is a critical diagnostic tool. The findings of colonic wall thickening (>4 mm) and megacolon (>6 cm diameter) on CT scan are hallmark indicators of severity [6,7]. In this case, radiology provided the definitive evidence of disease severity that compelled aggressive medical management, overriding the negative toxin assay.

While the lack of reflex molecular confirmation is a limitation in this case, the diagnosis of fulminant CDI was made clinically based on a positive GDH antigen in the setting of toxic megacolon and significant leukocytosis. Given that toxin EIA has a high false-negative rate, and the patient’s critical status precluded waiting for further confirmatory testing, empirical treatment was initiated. The subsequent resolution of toxic megacolon upon targeted CDI therapy further supports the diagnosis.

## Conclusions

This case serves as a critical reminder that a negative *C. difficile* toxin EIA does not exclude severe, life-threatening infection. Clinicians must maintain a high index of suspicion for fulminant CDI in patients with compatible symptoms, especially new or worsening abdominal distension, high-volume diarrhea, and systemic inflammation, irrespective of initial toxin test results. Clinical judgment, supported by timely radiological imaging, must guide the decision to initiate aggressive empirical therapy to prevent progression to toxic megacolon, colectomy, and death. The literature unequivocally supports treating the patient’s clinical syndrome, not the laboratory report. Most importantly, however, unnecessary PPI treatment should be deprescribed by a pharmacist or general practitioner as it is a known risk factor.
